# Contribution of BRCA1 and BRCA2 germline mutations to early onset breast cancer: a series from north of Morocco

**DOI:** 10.1186/s12885-020-07352-9

**Published:** 2020-09-07

**Authors:** Joaira Bakkach, Mohamed Mansouri, Touria Derkaoui, Ali Loudiyi, ElMostafa El Fahime, Amina Barakat, Naima Ghailani Nourouti, Jaime Martinez De Villarreal, Carlos Cortijo Bringas, Mohcine Bennani Mechita

**Affiliations:** 1 Biomedical Genomics and Oncogenetics Research Laboratory, Faculty of Sciences and Techniques of Tangier, University Abdelmalek Essaâdi, P.A: 416-Tangier, Tangier, Morocco; 2 Oncology Clinic Al AMAL of Tangier, Tangier, Morocco; 3 Functional Genomic Plateform, Units of Technical Support to Scientific Research, National Center of Scientific and Technical Research, Rabat, Morocco; 4 Genetracer Biotech Laboratory, Cantabria, Spain

**Keywords:** Breast cancer, Young women, BRCA1, BRCA2, Germline mutations, Genetic testing, Morocco

## Abstract

**Background:**

To date, the contribution of BRCA1/2 mutations in Moroccan early onset breast cancer patients remains unknown. Here we assess these genetic alterations for the first time in a cohort from North of Morocco.

**Methods:**

Thirty-three patients diagnosed with breast cancer at the age of ≤40 years were recruited irrespective of breast and/or ovarian cancer family history. Coding regions and intron-exon boundaries of BRCA1 and BRCA2 genes were sequenced from peripheral blood DNA using Ion Proton (Thermo Fisher Scientific) next generation sequencing platform.

**Results:**

Overall, five BRCA germline mutations were identified (15.1%). The frequency of mutations among patients with family history of breast cancer was 16.7%. Three mutations were found in BRCA1 (9%) and two within the BRCA2 gene (6%). These are three frameshift mutations (c.798_799del, c.2125_2126insA, c.5116_5119delAATA), one missense (c.116G > A) and one nonsense mutation (c.289G > T). The mutation c.5116_5119delAATA has a founder effect in North Africa. Moreover, one variant of unknown significance was identified in BRCA2 (c.4090A > G). Most BRCA mutations carriers (80%) had no family history of breast cancer.

**Conclusion:**

Our data do not support the hypothesis that BRCA mutations alone explain the higher frequency of breast cancer in Moroccan young women. The young age (≤40 years) for breast cancer diagnosis seems to be strongly predictive of BRCA mutation status in Moroccan patients. These results will help in decision making with regard to genetic counseling and testing in the national scale.

## Background

In Morocco, and other less developed countries, breast cancer occurs more frequently in young population. The young age for development of breast cancer often suggests a genetic predisposition especially germline mutations in BRCA1 and BRCA2 genes. These genetic alterations were shown to be involved in up to 12% of early-onset breast cancer (EOBC) cases in western countries [1]. It is well established that the frequency of these mutations differs according to inclusion criteria, screening methods, geographical location and ethnic origin. However, the prevalence of BRCA1/2 mutations is still unknown among Moroccan young breast cancer patients and it is still unclear whether these genetic factors may explain the higher rate of EOBC in Morocco.

The aim of the present study was to analyze BRCA1 and BRCA2 germline mutations in EOBC patients who were unselected for a family history. To the best of our knowledge, this is the first genetic study of its kind carried out in North of Morocco and the first study that used only young age as selection criteria irrespective of family history. This work is an important milestone in determining the prevalence of BRCA1/2 germline mutations in EOBC in the Moroccan population.

## Methods

### Study patients

From January 2010 to December 2015, a total of 82 young patients (≤ 40 years) with invasive breast carcinoma were referred to the Oncology Clinic AL AMAL in Tangier. Clinico-pathologic, prognostic and therapeutic features of the whole group were previously published [2]. Alive patients were invited to participate in the genetic study and were informed about potential implications of the test. All these patients were selected based on young age (≤ 40 years) irrespective of family history of breast and/or ovarian cancer. Thirty-four patients agreed to participate in the study (One patient withdrew consent for personal reasons). All recruited patients elected to receive their results.

Clinico-pathologic data was collected from medical records. Information about family history was completed during recruitment.

This study was approved by the Biomedical Research Ethics Committee in the Faculty of Medicine and Pharmacy in Rabat (CERB) and an informed written consent was obtained from all participants. Blood samples were obtained from each patient during the interview process. Results were treated with confidentiality and were not included in medical records.

### Genetic testing

Genomic DNA was isolated from 200 μL peripheral-blood using QIAamp DNA isolation kit and stored at − 20 °C. Mutational analysis was performed at GENETRACER BIOTECH Laboratory, Cantabria in Spain or at the National Center of Scientific and Technical Research, Rabat (Units of Technical Support to Scientific Research) in Morocco. All coding sequences of BRCA1 and BRCA2 genes and exon–intron boundaries were analyzed using the Ion Proton (Thermo Fisher Scientific) next generation sequencing platform. The NGS library was constructed by a multiplex polymerase chain reaction (PCR)-based assay (Ampliseq technology) using Oncomine BRCA Research Assay according to the manufacturer’s protocol. Reads were aligned to the human genome reference sequence 19 (hg19) using Torrent Browser 5.2 software and Ion Reporter 5.2. Exonic sequence analysis was performed with an average coverage greater than 4500X.

The results obtained with the Ion Reporter 5.2 software were filtered by eliminating all the alterations considered as common polymorphisms by the University of California Santa Cruz (UCSC) genome browser. All recorded variants were germline heterozygous mutations. The mutations obtained by this filtering process were verified in the UCSC genome browser and in various databases including EXAC, dbSNP, COSMIC, UMD, LOVD, BIC and ClinVar and also in the scientific literature. Finally, the results obtained were verified by the algorithm Sophia DDM™-BRCA-Ampliseq™-GL-IonProton™ (Sophia Genetics).

Patients with pathogenic or likely pathogenic variants were considered to be mutation carriers. Patients with normal sequencing results and those with variants of unknown clinical significance (VUS), benign or probably benign variants were considered as non-carriers.

## Results

The median age at diagnosis was 35 years (range: 28–40). More than half of patients (51.5%) were diagnosed before the age of 35 and 48.5% were aged 36–40 years old. Family history was unknown for one patient. A family history for breast cancer (1st or 2nd degree) was recorded for 18.8% of cases. Two patients had bilateral cancer. Almost one quarter of patients (24.2%) were presented at an advanced stage (Locally advanced: 21.2% and metastatic: 3%). Most patients had invasive carcinoma of no special type (NST) (93.9%), and 3% were diagnosed with lobular or mucinous carcinoma. 51.5 and 39.4% of patients had intermediate and high grade carcinomas, respectively and 30.3% of cases had triple negative tumors.

Among the 33 patients aged ≤40 years who were unselected for family history, five BRCA mutations were identified in five unrelated patients (15.1%). Three mutations were found in BRCA1 gene (9%) and two in BRCA2 (6%). Of the 17 patients who were aged ≤35 years, the frequency of BRCA1/2 mutations was 23.5%. Of the 16 patients aged 36–40 years, 6.2% were BRCA1/2 mutations carriers. When stratified by family history (1st or 2nd degree), 4 of 26 (15.4%) who were aged ≤40 with negative family history had BRCA mutations (3 BRCA1 and 1 BRCA2). In contrast, only 1 of 6 patients (16.7%) who were aged ≤40 with positive family history had BRCA mutations (1 BRCA2).

Of the three mutations identified on BRCA1 gene, two were located on exon 11 and one on exon 3. Of the two BRCA2 mutations, one was located on exon 11 and the other on exon 3. These include three frameshift mutations: BRCA1: c.798_799del (p.Ser267Lysfs), BRCA1: c.2125_2126insA (p.Phe709Tyrfs), BRCA1: c.5116_5119delAATA (p.Asn1706Leufs), one missense BRCA1: c.116G > A (p.Cys39Tyr) and one nonsense mutation BRCA2: c.289G > T (p.Glu97Ter). The mutation BRCA1: c.116G > A (p.Cys39Tyr) is a substitution of G with A at position 39 at exon 3 of the BRCA1 gene. The mutation BRCA1:c.2125_2126insA (p.Phe709Tyrfs) is an insertion of an A that produces an alteration in the reading frame and leads to a STOP codon in exon 10 of BRCA1. The mutation BRCA1: c.798_799del (p.Ser267Lysfs) is a deletion of 2 T bases that leads to the premature termination of protein synthesis at codon 285 of exon 10 of BRCA1. The mutation BRCA2: c.5116_5119delAATA (p.Asn1706Leufs) is a deletion of AATA that produces alteration in the reading frame and leads to a STOP codon in exon 11 of BRCA2. The mutation BRCA2: c.289G > T (p.Glu97Ter) is a G-base substitution by T leading to a premature STOP codon at exon 3 of the BRCA2 gene (Table 1).

**Table 1:** BRCA1 and BRCA2 mutations and unclassified variants

Variant	Exon	BIC Nomenclature	BIC entries^a^	Type	Protein	Clinical significance	BRCA exchange^a^
**NM_007294.3 (BRCA1):c.116G > A**	3	235G > A	5	Missense	NP_009225.1:p.Cys39Tyr	Likely Pathogenic	Not yet reviewed
**NM_007294.3(BRCA1):c.2125_2126insA**	11	2244insA	2	Frameshift	NP_009225.1:p.Phe709Tyrfs	Pathogenic	Pathogenic
**NM_007294.3 (BRCA1):c.798_799del**	11	917_918del	28	Frameshift	NP_009225.1:p.Ser267Lysfs	Pathogenic	Pathogenic
**NM_000059.3 (BRCA2)**: **c.289G > T**	3	516G > T	0	Nonsense	NP_000050.2:p.Glu97Ter	Pathogenic	Pathogenic
**NM_000059.3(BRCA2):c.5116_5119delAATA**	11	5344delAATA	1	Frameshift	NP_000050.2:p.Asn1706Leufs	Pathogenic	Pathogenic
**NM_000059.3(BRCA2):c.4090A > G**	11	4317A > G	0	Missense	NP_000050.2:p.Ile1364Val	VUS	Not Yet Reviewed
**NM_000059.3(BRCA2):c.6322C > T**	11	6549C > T	22	Missense	NP_000050.2:p.Arg2108Cys	Conflicting interpretations of pathogenicity	Not Yet Reviewed

^a^: Accessed 06/08/2019. *Abbreviations*: *VUS* Variants of Unknown Significance

All mutations have been identified only once. A single mutation was not previously reported in BIC, but has already been described in the literature BRCA2: c.289G > T (p.Glu97Ter). No patient presents simultaneously two mutations.

In addition to these mutations, one BRCA2 VUS was found BRCA2: c.4090A > G (p.Ile1364Val) and co-occurred with a likely pathogenic mutation BRCA1: c.116G > A (p.Cys39Tyr). Another variant BRCA2: c.6322C > T (p.Arg2108Cys) with conflicting interpretations of pathogenicity was also identified in BRCA2 (Table 1).

Most (80%) BRCA mutation carriers were aged ≤35 years, without 1st or 2nd degree breast cancer family history, had early-stage disease, and were diagnosed with unilateral breast cancer (4/5). All mutation carriers had NST carcinoma. Furthermore, 60% of our mutated patients had high-grade and triple negative tumors (3/5) (Table 2).

**Table 2:** Clinico-pathologic characteristics of BRCA mutation carriers

ID	Variant	Age (Interval)	Family History	Bilateral cancer	Stage	Histology	mSBR grade	Molecular subtype
1	BRCA1 c.116G > A + BRCA2 c.4090A > G	28–35	Aunt M, lung Aunt P, liver	No	Early	NST	3	TN
2	BRCA1 c.2125_2126insA	28–35	P cousin, BC	No	Early	NST	2	TN
3	BRCA1 c.798_799del	28–35	No	No	Early	NST	3	TN
4	BRCA2 c.5116_5119delAATA	36–40	M cousin, uterus	Yes	Early	NST	3	Luminal
5	BRCA2 c.289G > T	28–35	P aunt, BC P Aunt, Arm	No	LA	NST	2	Luminal
6	BRCA2 c.6322C > T	36–40	P cousin, unknown	No	Early	NST	2	Luminal

*Abbreviations*: *BC* Breast cancer, *LA* Locally Advanced, *M* Maternal, *NST* Invasive carcinoma of No Special Type, *P* Paternal, *TN* Triple negative

## Discussion

Overall, only few genetic studies aiming to analyze BRCA1/2 germline mutations among unselected young breast cancer patients have been reported [3–10]. Historically, the prevalence among women aged under 40 varies from 4.8 to 11.6% [6, 8, 9, 11]. Our frequency (15.1%) is significantly higher than these western data. This large difference could be explained by the increased sensitivity of our screening method (NGS), unlike those older series which used mostly indirect techniques for mutations screening such as heteroduplex, Single Strand Conformation Polymorphism (SSCP), Denaturing Gradient Gel Electropheresis (DGGE)... More recently, a large multi-center study recruiting 2733 cases from 127 hospitals in the United Kingdom (POSH study) and using a NGS platform reported a frequency of 12% [1]. Our mutation rate did not differ greatly from these data suggesting thus similar contribution in western and Moroccan EOBC. Based on these data and in light of the limitations of our work, it seems that our findings do not support the hypothesis that BRCA mutations alone may explain the higher incidence of EOBC in our population. Larger multicenter studies are warranted to verify our results.

Studies among unselected EOBC (< 40 years) from Middle East and North Africa (MENA) region showed disparate results with a BRCA mutation rate ranging from 0 to 26% [12–15] (Table 3). Fortunately, all these reports have sequenced both BRCA genes and also analyzed large genomic rearrangements, unlike our work. However, the limited size of most of these studies makes it difficult to draw firm conclusions about the contribution of BRCA alterations to EOBC in this world region. In Morocco, to the best of our knowledge, the current study is the first study for BRCA testing in unselected EOBC patients. Earlier reports have been drawn from unusual cancer-rich or cancer-free families [16–18] that are not representative of the family history profiles of women in the general population who develop breast carcinoma in young age.

**Table 3:** Published studies from MENA region analyzing BRCA mutations in early onset breast cancer

Study	No. patients	Family history	BRCA Analyzed regions	Screening method	BRCA mutation rate, Notes
**Morocco**					
Tazzite et al. [16]	4	SBC	BRCA1/2	Direct sequencing	25%
Laraqui et al. [17]	102	SBC	BRCA1	Direct sequencing	1%
Tazzite et al. [18]	28	Unknown FH^a^	BRCA1 (Ex2, 11a, and 11b)	Direct sequencing	12.2% ^b^
Jouali et al. [19]	15	FBC	BRCA1/2	NGS, direct sequencing	26.7%
The present study	33	Unselected BC	BRCA1/2	NGS	BC (≤40 y), unselected: 15.1% FBC (≤40 y): 16.7% SBC (≤40 y): 15.4% BC (≤35 y): 23.5% BC (36–40 y): 6.2%
**Algeria**					
Uhrhammer et al. [20]	51	SBC	BRCA1	Direct sequencing, MLPA	9.8%
Cherbal et al. [21]	52	FBC	BRCA1/2	HRM, Direct sequencing, MLPA	13.5%
Henouda et al. [13]	40	Unselected BC	BRCA1/2	Direct sequencing, MLPA	20%
**Tunisia**					
Mahfoudh et al. [22]	7	FBC	BRCA1	Direct sequencing	42.9%
Riahi et al. [23]	4	FBC	BRCA1/2	Direct sequencing	0%
**Egypt**					
Ibrahim et al. [24]	39 15	FBC SBC	BRCA1/2	SSCP (BRCA1: Ex2, 8, 13, 22; BRCA2: Ex9) Heteroduplex, direct sequencing	FBC (≤45 y): 89.7%
**Lebanon**					
El Saghir et al. [12]	148 102	Unselected BC FBC	BRCA/2	Direct sequencing, MLPA	BC (≤40 y), unselected: 6% FBC (≤40 y): 10.8% SBC (≤40 y): 1.4% FBC (41–50 y): 5.3% FBC (> 50 y): 3.7%
**Iran**					
Yassaee et al. [25]	83	Unselected BC	BRCA1 (Ex2,3,5,11, 13 and 20) BRCA2 (Ex9,10,11, 17,18 and 23)	PTT, SSCP	6.02% FBC: 28.6% SBC: 1.5%
Pietschmann et al. [26]	41	FBC	BRCA1/2	Direct sequencing, Semi-quantitative multiplex PCR (BRCA1)	9.8% LR: 0%
Keshavarzi et al. [27]	49 36	SBC FBC	BRCA1/2 (Except BRCA1:Ex1 and 4; and BRCA2: Ex1)	Direct sequencing	Global mutation rate: 11.8%
Yassaee et al. [28]	254	FBC	BRCA1/2	SSCP, PPT, MLPA, and direct sequencing	Global mutation rate: 18% MS: 39.1% Indels: 15.2% LR: 6%
Ebrahimi et al. [15]	NA	Unselected BC	BRCA1/2	NGS	0%
**Jordan**					
Abdel-Razeq et al. [14]	51	Unselected BC	BRCA1/2	Comprehensive BRAC Analysis® and BRAC Analysis® Rearrangement Test (BART)	Global mutation rate: 26% FBC: 32.5% SBC: 0% TN: 50%
**Syria**					
Khalil et al. [29]	32 18	FBC SBC	BRCA1 (Ex2) BRCA2 (Ex11)	Direct sequencing	15.6% 0% No association between age (≤40 y) and exon 11 mutations
**Palestine**					
Hamameh et al. [30]	79 186	FBC SBC	BRCA1/2	NGS	16.5% 2.7%
**Saudi Arabia**					
Alhuqail et al. [31]	108	Unselected BC	BRCA1/2	NGS	8.3% No correlation between age and mutation status
Abulkhair et al. [32]	66	FBC	BRCA1/2	NGS MLPA	BRCA1: 9.3% No association between age and mutation status

^a^: Authors assumed that these cases cannot be precisely defined as sporadic, and were most likely correlated with a family history; ^b^: This mutation rate included also FBC cases

*Abbreviations*: *BC* Breast Cancer, *Ex* Exon, *FBC* Familial Breast Cancer, *HRM* High Resolution Melting, *LR* Large Rearrangement, *MLPA* Multiplex Ligation-dependent Probe Amplification, *MS* Missense Mutations, *NA* Not Available, *NGS* Next generation Sequencing, *PPT* Protein Truncation Test, *SBC* Sporadic Breast Cancer, *SSCP* Single Strand Conformation Polymorphism

It has been suggested that BRCA1 and BRCA2 genes contribute equally to EOBC. We described a BRCA1/BRCA2 ratio of 1.5 (BRCA1 mutations: 9% and BRCA2 mutations: 6%), consistent with that reported in POSH study and other large population-based studies [1, 33, 34].

The BRCA1:c.116G > A (p.Cys39Tyr) mutation is classified as pathogenic in Clinvar with a pending classification in BIC. It is a recurrent mutation in Slovenia [35–37]. It was also found in North eastern Italy [38], in India [39], but never been reported in Morocco. This mutation affects the homologous recombination, leads to centrosome amplification, and may affect the formation of the BARD1/BRCA1 heterodimer [40]. This mutation was simultaneously present with a VUS, BRCA2:c.4090A > G (p.Ile1364Val), suggesting that this latter is a neutral variant. BRCA2:c.4090A > G has already been reported in the Moroccan population [16, 41]. In silico analysis with PolyPhen suggests lack of pathogenicity: PolyPhen-2 v2.2.2r398--HumDiv: Benign with a score of 0.005 (sensitivity: 0.97; specificity: 0.74); HumVar: Benign with a score of 0.003 (sensitivity: 0.98; specificity: 0.26). In silico analysis with NNSPLICE, MaxEntScan and Splice Site Finder show the creation of a donor site with a possible splicing effect.

The BRCA1:c.2125_2126insA (p.Phe709Tyrfs) mutation is a recurrent mutation in the French-Canadian population [42–46]. It has been also found in the United Kingdom [1]. More recently, it has been reported in Algeria in three young patients aged 33, 34 and 38 years respectively, all with triple negative cancer and a family history of breast and/or prostate cancer [47].

The BRCA1:c.798_799del (p.Ser267Lysfs) mutation has been detected in four Hispanic patients [48]. It was also identified in two Tunisian and Algerian families with a common haplotype to all carriers of this mutation [20], suggesting the presence of the first non-Jewish founder effect in North Africa. It was later identified in four Algerian patients [21, 47], three Moroccan women [16, 17] and five Tunisian women [22], all with a family history of breast or ovarian cancer and aged between 34 and 44 years.

The BRCA2:c.289G > T (p.Glu97Ter) mutation is not mentioned in BIC, but already reported in the literature. It has been identified in one patient in central Italy [49], in France [50] and in Korea [51].

The BRCA2:c.5116_5119delAATA (p.Asn1706Leufs) mutation is reported only once in BIC with a Caucasian ethnic origin. It was detected for the first time in northwestern Spain in Castilla y León [52] and has been described as a recurrent founder mutation in this region. Interestingly, it has been associated with EOBC (mean age 37.4 years; *p* = 0.033) [53]. Overall, this mutation has been identified in a group of eight independent Spanish families [53–55], and also in two Korean patients [56].

In summary, according to our data, it seems that there is a great heterogeneity in the spectrum of BRCA1/2 mutations in our population study. More specifically, this spectrum appears to be shared with other Mediterranean populations, which can be explained by the geographic location of our country.

The vast majority of BRCA1/2 mutations identified in our work occur in a non-family context (80%). This may be due to low penetrance of BRCA mutations due to genetic or environmental risk modifying factors. Another possible explanation is the limited size of families of mutation carriers, or a possible preponderance of males or normal alleles rather than segregated altered alleles. The presence of a memory bias is also not excluded since family history data were collected retrospectively after the cancer diagnosis and this information was not verified.

Young age of onset for breast cancer is a testing criterion in already published recommendations with a disparity in the age thresholds between guidelines (National comprehensive cancer network: ≤45 years, and Saint Gallen consensus: ≤35) [57, 58]. However, in Morocco the prevalence of BRCA1/2 mutations is yet to be well defined, which adds further challenge in tailoring international guidelines to the national context. We have shown that the frequency of mutations among young patients who were unselected for family history (15.1%) exceeds the probability threshold required for BRCA genetic testing (≥10%). Moreover, the presence of a family history seems to raise only slightly the mutations rate (16.7%) and a significant proportion of mutation carriers were sporadic cases (80%). Hence, limiting BRCA testing to high-risk women could lead to miss a significant number of mutations. In light of these data, we suggest that young age alone is a sufficient criterion to indicate a systematic genetic screening and that the age limit of ≤40 years is an adequate threshold for our population. This remains to be further investigated.

The present study shows some limitations. The sample size of our analyzed series (*n* = 33) and the number of detected BRCA1/2 mutations (*n* = 5) were small, thus we could not provide conclusive data about the prevalence and spectrum of BRCA1/2 mutations in young breast cancer patients from North of Morocco. The time interval between patient’s recruitment and molecular analysis may be another limitation for our study. Alive patients who were referred to our Clinic between January 2010 to December 2015 were recruited and molecular analysis was carried out in 2017 (33 patients from 82 initially identified). The patients “selection” by delayed molecular analysis can change some characteristics such as family history, metastatic cancer and triple negative breast cancers rates (26% in the initial group *Vs.* 18.8% in the analyzed group, 12.2% *Vs.* 3%, and 23% *Vs.* 30.3%, respectively) [2]. Lastly, our analysis was restricted to BRCA1/2 mutations and did not include large rearrangements nor copy number variations.

## Conclusion

Our frequency of BRCA1/2 germline mutations does not differ greatly to Western data. These genetic alterations alone seem not to explain the higher incidence of breast cancer in young Moroccan women in contrast to what was expected. The implication of other gene mutations such as PALB2, TP53 should be investigated. Copy number variations were highly biologically relevant in breast cancer and their study would be of interest as some of them were suggested to contribute to the aggressive nature of tumors arising in younger patients.

Our data suggest that young age (≤ 40 years) irrespective of family history is a sufficient criterion for systematic genetic screening for Moroccan young breast cancer patients, following the western recommendations for BRCA1/2 genetic testing.

Finally, our study could not provide conclusive data due to some limitations including possible “patients selection” by delayed molecular analysis (retrospective analysis).Larger and prospective studies are warranted to confirm our findings.

## Data Availability

All the variants found in this study were deposited in the LOVD database: #0000643111(https://databases.lovd.nl/shared/variants/0000643111#00003478); #0000643125 (https://databases.lovd.nl/shared/variants/0000643125#00003478); #0000643126 (https://databases.lovd.nl/shared/variants/0000643126#00003478); #0000643127 (https://databases.lovd.nl/shared/variants/0000643127#00003479); #0000643128 (https://databases.lovd.nl/shared/variants/0000643128#00003479); #0000643129 (https://databases.lovd.nl/shared/variants/0000643129#00003479); #0000643130 (https://databases.lovd.nl/shared/variants/0000643130#00003479).

## Abbreviations

BIC: Breast cancer Information Core
DGGE: Denaturing Gradient Electrophoresis
EOBC: Early Onset Breast Cancer
MENA: Middle East and North Africa
MLPA: Multiplex Ligation Probe Amplification
NGS: Next Generation Sequencing
NST: Invasive carcinoma of no special type
SSCP: Single-Strand Conformation Polymorphism
UCSC: University of California Santa Cruz
VUS: Variant of Unknown Significance
